# Can Previous Associations of Single Nucleotide Polymorphisms in the *TLR2*, *NOD1*, *CXCR5*, and *IL10* Genes in the Susceptibility to and Severity of *Chlamydia trachomatis* Infections Be Confirmed?

**DOI:** 10.3390/pathogens10010048

**Published:** 2021-01-07

**Authors:** Jelmer B. Jukema, Bernice M. Hoenderboom, Birgit H. B. van Benthem, Marianne A. B. van der Sande, Henry J. C. de Vries, Christian J. P. A. Hoebe, Nicole H. T. M. Dukers-Muijrers, Caroline J. Bax, Servaas A. Morré, Sander Ouburg

**Affiliations:** 1 Laboratory of Immunogenetics, Department Medical Microbiology and Infection Control, Amsterdam UMC, Location AMC, 1100 DD Amsterdam, The Netherlands; j.b.jukema@amsterdamumc.nl (J.B.J.); bernice.hoenderboom@rivm.nl (B.M.H.); samorretravel@yahoo.co.uk (S.A.M.); 2 Epidemiology and Surveillance Unit, Centre for Infectious Disease Control, National Institute for Public Health and the Environment, 3720 BA Bilthoven, The Netherlands; birgit.van.benthem@rivm.nl (B.H.B.v.B.); mvandersande@itg.be (M.A.B.v.d.S.); 3 Institute of Tropical Medicine, Department Public Health, 2000 Antwerp, Belgium; 4 Global Health Julius Centre for Health Sciences and Primary Care, University Medical Centre Utrecht, 3508 AB Utrecht, The Netherlands; 5 Amsterdam UMC, University of Amsterdam, Department of Dermatology, Amsterdam Institute for Infection and Immunity (AI&II), Location Academic Medical Centre, 1100 DD Amsterdam, The Netherlands; h.j.devries@amsterdamumc.nl; 6 Department of Infectious Diseases, STI Outpatient Clinic, Public Health Service, 1018 WT Amsterdam, The Netherlands; 7 Department of Sexual Health, Infectious Diseases and Environmental Health, South Limburg Public Health Service (GGD South Limburg), 6160 HA Geleen, The Netherlands; Christian.Hoebe@ggdzl.nl (C.J.P.A.H.); Nicole.Dukers@ggdzl.nl (N.H.T.M.D.-M.); 8 Department of Social Medicine and Medical Microbiology, Care and Public Health Research Institute (CAPHRI), Maastricht University Medical Centre (MUMC+), 6200 MD Maastricht, The Netherlands; 9 Department of Obstetrics and Gynaecology, Amsterdam UMC, Location AMC, University of Amsterdam, 1100 DD Amsterdam, The Netherlands; c.j.bax@amsterdamumc.nl; 10 Institute for Public Health Genomics (IPHG), Department of Genetics and Cell Biology, Research School GROW (School for Oncology & Developmental Biology), Faculty of Health, Medicine & Life Sciences, University of Maastricht, 6229 ER Maastricht, The Netherlands

**Keywords:** *Chlamydia trachomatis*, single nucleotide polymorphism, SNP, susceptibility, severity

## Abstract

Clear inter-individual differences exist in the response to *C. trachomatis* (CT) infections and reproductive tract complications in women. Host genetic variation like single nucleotide polymorphisms (SNPs) have been associated with differences in response to CT infection, and SNPs might be used as a genetic component in a tubal-pathology predicting algorithm. Our aim was to confirm the role of four genes by investigating proven associated SNPs in the susceptibility and severity of a CT infection. A total of 1201 women from five cohorts were genotyped and analyzed for *TLR2* + 2477 G > A, *NOD1* + 32656 T −> GG, *CXCR5* + 10950 T > C, and *IL10* − 1082 A > G. Results confirmed that *NOD1* + 32656 T −>GG was associated with an increased risk of a symptomatic CT infection (OR: 1.9, 95%CI: 1.1–3.4, *p* = 0.02), but we did not observe an association with late complications. *IL10* − 1082 A > G appeared to increase the risk of late complications (i.e., ectopic pregnancy/tubal factor infertility) following a CT infection (OR = 2.8, 95%CI: 1.1–7.1, *p* = 0.02). Other associations were not found. Confirmatory studies are important, and large cohorts are warranted to further investigate SNPs’ role in the susceptibility and severity of a CT infection.

## 1. Introduction

CT is the most diagnosed bacterial sexually transmittable infection (STI) worldwide [1], with an estimated 127 million new infections each year [2]. In the Netherlands, approximately 60,000 new infections occur on a yearly basis [3]. In women, an estimated 70–80% of the infections are asymptomatic [4]. These women are thus at risk to remain untreated; leaving them prone to late complications such as pelvic inflammatory disease (PID), ectopic pregnancy (EP), and tubal factor infertility (TFI) [1,5]. Estimating the individual risk of late complications is complicated by interpersonal differences in susceptibility, course, and outcome of the infection. These differences in women can at least to some extent be explained by bacterial factors (e.g., virulence, load), environmental factors (e.g., co-infection, microbiome), and host factors (e.g., immunogenetic differences between individuals, (sexual) risk behavior) [6,7].

Since CT is assumed to be an important cause of tubal pathology [8], subfertile women in the Netherlands who attend a fertility specialist are tested with a chlamydia antibody test (CAT), which can identify a past infection. If the CAT is positive, a hysterosalpingogram (HSG) is performed to examine the tubes and if the HSG is indicative for tubal pathology, a laparoscopy, which is the golden standard, follows. However, since the CAT is designed to detect a past infection and not to identify tubal pathology, it has a suboptimal predictive value for finding tubal pathology. This may lead to incorrect triage and thus to unnecessary tubal imaging. These invasive tests are uncomfortable, come with health hazards, and are expensive. Therefore, there is a need for more specific markers to identify increased risk for tubal pathology.

In addition to serology markers, host genetics could be important in the risk for tubal pathology. A considerable part of the interpersonal differences in responding to a CT infection can be explained by host genetics. A twin study has suggested that almost 40% of the difference in the immunological response to CT infection is based on host genetics [9]. A large number of Single Nucleotide Polymorphisms (SNPs) has been linked to differences in the susceptibility to and severity of a CT infection [10]. The most relevant SNPs for CT are in intra- and extra-cellular pathogen recognition receptors (PRRs), and in cytokines and chemokines involved in and modulating the immune response after infection with CT [11]. Some SNPs result in an enhanced risk for infection or complications after CT infection, while others lower the risk for infection or complications. Hence, a proposed way of improving current fertility workup is the development of a tubal-pathology predicting algorithm based on host genetics in combination with serology [12].

Four well-described SNPs that have previously been associated with the outcomes of a CT infection are: *TLR2* +2477 G > A (rs5743708), *NOD1* + 32656 T −> GG (rs6958571), *CXCR5* + 10950 T > C (rs3922), and *IL10* − 1082 A > G (rs1800896). TLR2 has been shown to play an important role as a mediator in the innate immune response to a CT infection. It has also been shown to be important in the early production of inflammatory mediators and the development of chronic inflammatory pathology [13]. Verweij et al. found that *TLR2* + 2477 *A provided an increased risk for the development of tubal pathology in CT positive women (OR 17.5) [14], although the study group was rather small. CT seropositive women carrying the *NOD1* GG insertion had a more than double increased risk of tubal pathology (OR: 2.25; 95%CI: 1.08–4.67, *p* = 0.04) [15]. NOD1 normally functions as an intracellular pattern receptor, but the GG insertion creates a stop codon, thus impairing the functioning of the gene. In contrast to the *TLR2* and *NOD1* mutations, the SNP in the *CXCR5* gene was protective. *CXCR5* CC had a large protective effect for CT positive women (OR: 0.1, 95%CI: 0.04–0.5, *p* = 0.002) against developing tubal pathology [16]. Furthermore, finally, the A allele of the *IL10* − 1082 G > A SNP, which resides in the promotor region of this immunosuppressive cytokine, protected against the development of severe tubal damage [17].

The aim of this study is to build the evidence base for the role of human genes in CT infection, and assess to what extent the earlier described associations of SNPs in four genes in the susceptibility to and severity of a CT infection using clinically well-defined cohorts could be confirmed.

## 2. Results

### 2.1. DNA Isolation and SNP Determination

SNP determination for cohorts 1–4 was done by LGC, UK. The Genotyping success rate for the four SNPs in these cohorts ranged from 77.6% to 99.1%. For cohort 5, 178 samples were isolated and genotyped in-house. SNP determination of all four SNPs was successful for 162 DNA samples (91%). All SNPs were present in the cohorts, with TLR2 having the lowest minor allele frequency of 6.2%

### 2.2. Susceptibility to CT Infection

The genotype distribution based on CT status is shown in Table 1. Genotype distribution in cohort 3 differed significantly from genotype distribution in cohort 1 and 2. Hence cohort 1 and 2 (N = 304) were combined for this analysis, and cohort 3 (N = 707) was analyzed separately. A difference in the genotype distributions of *IL10* -1082 A>G was found in cohort 3 (*p* = 0.05). For women carrying *IL10* *G risk of CT infection was slightly lower compared to women who were homozygous wildtype, but this did not reach statistical significance (OR 0.6, 95%CI: 0.3–1.2, *p* = 0.14). No statistically significant associations were observed (all *p* > 0.3) between the three other studied SNPs and the susceptibility to a CT infection. This also remained unchanged in the sensitivity analysis, in which a fraction of cohort 1 for *NOD1* was excluded. The area under the curve (AUC) of the regression models for the susceptibility analysis in cohort 1 and 2 was 0.52 (95%CI: 0.45–0.59), and 0.57 (95%CI: 0.50–0.63) for cohort 3.

### 2.3. Severity of CT Infection

In Table 2, the SNP genotype data are given used for the severity analyses to CT infection.

I: We observed that CT positive women with the *NOD1* GG insertion were more likely to have a symptomatic course of infection (OR: 1.9, 95%CI: 1.1–3.4, *p* = 0.02) as compared to an asymptomatic infection. This association remained unchanged in the sensitivity analysis (Appendix A Table A2 and Table A4). Carriage of *TLR2* + 2477*A approached significance when assessing CT positive women with a symptomatic course of infection compared to CT positive women without one (OR: 2.6, 95%CI: 0.8–8.0, *p* = 0.10). The other two SNPs were not statistically associated with the severity of infection (Table 3).

II: In women with and without late complications from cohort 4 and 5 we did not observe significant differences in SNP distributions, although *IL10* approached statistical significance. CT positive women carrying *IL10* GG had a marginally, but not statistically significant, increased risk for developing complications after a CT infection (OR: 1.9, 95%CI: 1.0–3.6, *p* = 0.07) (Table 3). When comparing CT positive women with ectopic pregnancy/TFI to the fertile CT controls (excluding PID cases) the women carrying *IL10* GG had a significant higher risk of developing late complications (OR = 2.8, 95%CI: 1.1–7.1, *p* = 0.02). No associations for the other SNPs were found in this analysis.

**Figure 1:**
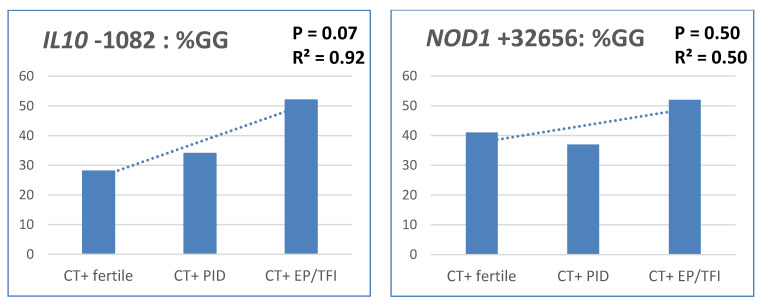

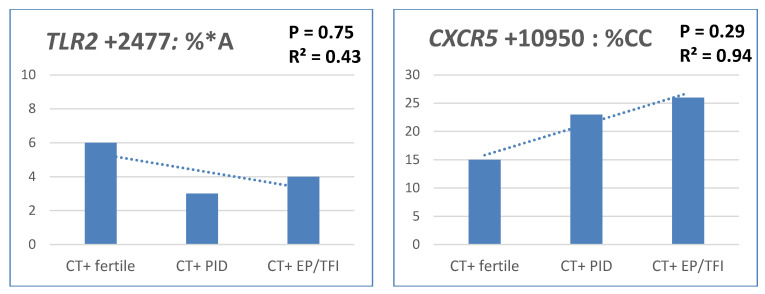
Trend analysis of Cohorts (**4**) and (**5**) Comparing genotype distributions between women based on increased severity: (**1**) Fertile CT positive women. (**2**) CT positive women with PID. (**3**) CT positive women with ectopic pregnancy and/or tubal factor infertility. Percentage of genotypes as part of total. Abbreviations: CT+, Chlamydia trachomatis positive; PID, pelvic inflammatory disease; EP, ectopic pregnancy; TFI, tubal factor infertility.

III: Trend analysis using cohorts 4 and 5 are shown in Figure 1. *IL10* GG showed an R^2^ of 0.92 (*p* = 0.07). Carriage of the GG genotype was more common among women with increased severity: 28% for fertile CT positive women to 34% for CT positive with PID to 52% for CT positive women with ectopic pregnancy/tubal factor infertility. No such association was observed for the other SNPs in this analysis.

IV: The AUC for the regression model performed on all four SNPs comparing symptomatic vs. asymptomatic in cohort 1 and 2 was 0.60 (95%CI: 0.52–0.68). The same score of 0.60 was obtained in the analysis of cohort 4 and 5 (95%CI: 0.52–0.69). After correcting results for the other investigated SNPs similar results as to the uncorrected data were found (Table 3 and Table 4).

## 3. Discussion

The aim of this study was to add to the current evidence base on the role of human genes in CT infection by assessing whether we could confirm the previously observed role of SNPs in the genes *IL10*, *NOD1*, *TLR2*, and *CXCR5* in the susceptibility to and severity of a CT infection. These four genes are all involved in detecting micro-organisms and starting the inflammatory response. Disease pathology is based on the functionality of these four genes linked to the SNPs studies. Meaning the severity of infection is genetically based in individuals. The novelty of this study is confined to confirming already proven associations with new and more data. Confirmation of these previous associations would be one step further in the direction of using these SNPs as a genetic part of a tubal pathology predicting algorithm. This algorithm will aim to differentiate between women likely to have fertility problems due to CT infections and women without increased risk. Saving women without increased risk unnecessary tubal imaging will save them an uncomfortable, expensive, invasive test with health hazards. In addition, earlier tubal imaging of women with a genetically very high risk of infertility due to CT can save a couple trying to become pregnant in vain. On the other hand, women without CT antibodies (and thus a low risk to tubal pathology) but with a high genetic risk profile should be investigated in more detail instead of trying to become pregnant for a year longer. This is due to two potential effects: (1) Loss of antibodies to CT and (2) tubal pathology due to other STDs like Neisseria, for which the SNP algorithm potentially also works.

The viability of such a tubal pathology predicting algorithm is still subject to scientific debate. Earlier attempts in other complex diseases to use polygenic risk scores, which were based on small numbers of highly significant SNPs identified from GWA studies, achieved only limited predictive value [18]. However, this algorithm will not only be based on genetics. Current machine learning methods allow for unprecedented pattern detection in both genetics and other factors. Other factors could possibly include the interaction between genetic variants and different disease serovars, amount of infections, co-infections, treatment (failure), age, birthplace, how positive the CAD test turns out, and sexual behavior. For now, this manuscript focuses solely on the genetic component and, more specifically, the confirmation of four previously proven SNP’s. If such a mentioned algorithm will never be created, or if it does not employ host genetics, at the very least, this study aids in further uncovering host factors driving ascension and pathology.

An association with susceptibility for CT infections was previously found for *NOD1* + 32656 T > GG but could not be confirmed in the current study. In contrast, the association of *IL10* − 1082 A > G with susceptibility was not earlier investigated, where we observed a protective effect. However, when comparing carriers of the mutation vs. homozygote wild-type, significance was lost (aOR 0.6, *p* = 0.15).

In the severity analysis, we confirmed the role for *NOD1* + 32656 T > GG; *NOD1* + 32656 *GG associated with a twofold higher risk of a symptomatic course of CT infection. CT Positive women with *IL10* − 1082 GG had an almost threefold higher odds ratio for developing late complications (i.e., EP/TFI) compared to CT positive females with the A* genotype. When including PID in the analysis, as well as in trend analysis comparing fertile CT positive women to CT positive women with PID to CT positive women with EP/TFI, a near significant (*p* = 0.07) association was found for *IL10* − 1082 GG. The role of the SNPs *TLR2* + 2477 G > A and *CXCR5* + 10950 T > C in the severity of infection could not be confirmed in this study.

NOD1 normally functions as an intracellular pattern receptor and is capable of triggering the host’s innate immune signaling pathways. This results in the production of pro-inflammatory cytokines, which are a vital part of the host defense against CT [19]. However, *NOD1* + 32656 T > GG creates a stop codon, thus impairing the functioning of the gene and the host defense. Branković et al. found a protective effect for *NOD1* + 32656 *GG (OR 0.52; 95%CI: 0.32–0.83, *p* = 0.006) in the susceptibility to infection [15] while we did not find an association between this SNP and the susceptibility to a CT infection. A plausible explanation for this difference is that Branković used a more strict definition. Our definition is positivity for CT DNA, while his research only used women who were both CT DNA and CT IgG positive, compared to women negative for both. When assessing the severity of a CT infection, Branković’s study found that carrying the *NOD1* GG insertion increased the risk of tubal pathology (OR: 2.25; 95%CI: 1.08–4.67, *p* = 0.04). When comparing CT-positive women without symptoms to CT-positive women with symptoms to CT-positive women with TFI, Branković found an increasing trend in carriage of the GG allele (*p*-trend: 0.0003). While we could confirm the *NOD1* GG insertion association with a symptomatic course of a CT infection, we did not find an association between the GG insertion and late complications. Concerning the OR for late complications, an important difference between Branković’s research and ours is that Branković reported on CT positive women diagnosed with TFI, and we, in this analysis, did not take the CT status into account. The difference in results in trend analysis is probably also due to definition differences. Branković compared asymptomatic women to symptomatic women to women with TFI (all CT positive), while we compared fertile women to women with PID to women with EP/TFI (all CT positive).

Our result of *IL10*−1082 A > G being a risk SNP is contrary to our hypothesis, which was based on research done by Ohman et al. They showed that the A allele was significantly associated with increased disease severity after CT infection [17]. In addition, other research indicated the AA genotype as a risk factor for Chlamydial TFI [20]. The GG genotype was found in 41.1% of our cases, while Ohman had found 19.8%. The AA genotype was found in 24.1% and 29.2% for our study and Ohman’s study, respectively, a remarkable difference in genotype distributions for cases in different populations. A possible explanation could be that the genotype distribution of the *IL-10* − 1082 SNP in Finland is quite different compared to our West-European population [17]. The results we found for *IL10* are seemingly contradicting when looking at the protective effect for *IL10* − 1082 *G in the susceptibility to CT infection versus the risk and role of the *IL10* − 1082 GG genotype in the severity of a CT infection. An explanation could be that these are clearly two different stages in complications of CT infections. It has been shown that *IL10* suppresses the inflammatory functions of macrophages, NK cells, dendritic cells, Th1, Th2, and B lymphocytes by regulating the expression of interferon-γ, tumor necrosis factor-α, major histocompatibility complex class II antigens, and co-stimulatory molecules, making it one of the most important regulatory factors [21,22,23]. The *IL10*-1082 SNP, which resides in the promotor region, forms three haplotypes with two other SNPs in this promotor region: −819 C > T and −592 C > A. The haplotypes formed are: GCC, ACC, and ATA. ACC and ATA are generally linked with low cytokine production; GCC is linked with a high IL10 production [24,25]. However, results differ per study, and also the reverse has been suggested [26,27]. For our study, it could be hypothesized that if GG is the genotype with low IL10 production, then the lack of suppression (especially of interferon-γ [25]) will upregulate the host defense against intracellular infections, clearing the infection at an early stage. However, if the upregulated immune system is unable to clear the infection, it might be stimulated too much, resulting in enhanced inflammation and tissue damage and thus increasing the chance of episodes of PID and potentially subsequent tubal scarring. The absence of an association between susceptibility and *TLR2* + 2477 G > A could be explained by earlier studies, which found it only associated with haplotype combinations [14]. The haplotypes with an increased risk of infection were heterozygous (GA) or homozygous (GG) for +2477 SNP [14]. In our study, *TLR2* approached significance when comparing CT positive women with a symptomatic course of infection to CT positive women with an asymptomatic course of infection. In the original research, using cohort 1, no such near-significant value was found [14]. In the study by Verweij et al. *TLR2* +2477 *A was also more frequently present in patients with tubal pathology (19.2%) compared to women without tubal pathology (0%, *p* = 0.015) [14]. The mechanism explaining these associations might be a lowered responsiveness to lipoproteins by the mutation [28], making it more difficult to recognize the CT particle.

CXCR5 has mainly been studied in mice thus far, in which it appears to regulate CD4- and natural killer T-cells [16]. The *CXCR5* + 10950 CC genotype of this chemokine receptor has been found to protect CT positive women with an OR of 0.1 of developing tubal pathology. [16] In our study, this finding could not be confirmed. Consistent with the previous findings [16], it did not associate with altered susceptibility to a CT infection.

Several limitations can be noted for this study. First, even though the women with late complications were selected out of large cohorts, the total number, in the end, is still relatively small. Second, for power purposes, we decided to include as many people as possible and thus confirmed earlier results using partly the same participants. However, sensitivity analysis yielded similar results. This sensitivity analysis was done using only the women who had never been tested before for these SNPs. Alternatively, if this method was not possible, comparing never before used cases with already analyzed controls. Third, the SNP distribution in cohort 3 did not match the SNP distribution of cohort 1 and 2 (*p* for the difference between groups ≤ 0.05). This means we needed to assess the susceptibility in cohort 1 and 2 combined and 3 separately, resulting in an unexpected loss of power. The difference between the SNP distributions could be the different geographical locations of cohort 3 inside the Netherlands. A study employing whole-genome sequencing to investigate variation and population structure in the Netherlands identified non-random sharing of rare mutations within and across provinces [29]. In addition, it used principal component analysis of common SNPs (frequency > 5%) to show a subtle substructure along a north-south gradient in the Netherlands [29]. Fourth, the definition of PID is a difficult one and as can be seen in Figure 1 the PID group does not always follow the same expected trend when trend analyzing with increasing severity are performed, even though CT is linked to PID. Fifth, we could not correct for co-infections as we do not have this data. Therefore, we cannot be absolutely certain that late complications are not caused by co-infections like Gonorrhea. However, this prevalence in Holland is low [3]. Considering cohort 1–3, a limitation is that susceptibility was only measured at one point in time.

We could not confirm all prior findings. This shows on one hand that confirmatory studies are of high importance and on the other hand that larger studies to further investigate these four, and other SNPs, are warranted. The ultimate goal of these studies is to determine the potential of these SNPs as a genetic component of a tubal-pathology prediction algorithm among CT positive women. The aim of the algorithm is twofold. First, to minimize the number of infertile women who try to become pregnant naturally, while actually IVF is indicated. Second, to reduce the number of fertile women unnecessarily undergoing a laparoscopy. In conclusion, our research does not exclude that genetics may in part be associated with the susceptibility and severity of CT infections, however, there is insufficient evidence to justify the routine determination of the genetic signature of the four studies SNP’s in clinical practice yet. More research for these SNP’s and other genetic variations to provide more insight seems needed.

## 4. Materials and Methods

### 4.1. Studied Cohorts

A total of 1201 women from 4 different STI and one late CT complication cohorts were included in this study, which aimed to confirm the role of SNPs in the 4 genes, *IL10*, *NOD1*, *TLR2*, and *CXCR5*, in the susceptibility to and severity (which was separated in symptomatic course and late complications) of CT infection in women. Characteristics of the 5 cohorts are listed in Table 5. Cohorts 1–3 were used to test susceptibility and cohorts 1, 2, 4, and 5 to test the severity of CT infection. Cohort 3 only contained information about CT status, not severity of infection, hence, it was not included in the severity analysis. Cohorts 4 and 5 consisted only of CT positive women and were, therefore, not included in the susceptibility analyses. From all cohorts, only women from West-European ethnicity (i.e., Dutch, British, Austrian, Belgian, German, Irish, or Luxembourgish) were included. From all samples of included women, DNA was isolated for SNP determination, as listed in Table 5. Sample material was either serum, a buccal swab, a vaginal swab, urine, or PBMC (peripheral blood mononuclear cell).

Since SNP analysis requires large datasets, a percentage of women who had also already previously been tested for these SNPs were included to maximize the amount of data. For 3 out of 4 SNPs (*NOD1* + 32656 T −> GG, *TLR2* +2477 G > A, and *CXCR5* + 10950 T > C), the susceptibility to a CT infection had previously been studied using (part of) cohort 1. In these previous studies, *NOD1* and *TLR2* used a different outcome, i.e., including CT serology positivity. [14,15,16] For the current confirmation study, 2 cohorts have been added, making the percentages of overlap between the current and the previous studies 11%, 19%, 19%, and 0% for *NOD1*, *TLR2*, *CXCR5,* and *IL10*, respectively. For severity, 2 SNPs (*NOD1* + 32656 T −>GG and *TLR2* + 2477 G > A) in part used the same cohorts as the original research; cohort 1 was previously used for symptomatology assessment in *NOD1* (26% overlap) (14) and *TLR2* (47% overlap) (13). Furthermore, 21% of cases (and 0% of controls) for late complications matched with the original paper for NOD1 (14). The majority (86%) of SNP determinations were done on women not previously tested for these SNPS.

### 4.2. SNP Determination

The isolated DNA samples were used to determine the SNPs *TLR2* + 2477 G > A (rs5743708), *NOD1* + 32656 T −> GG (rs6958571), *CXCR5* +10950 T>C (rs3922), and *IL10* − 1082 A > G (rs1800896). The SNPs were genotyped either at LGC in the United Kingdom (cohort 1–4) or in our own laboratory of Immunogenetics VUmc, The Netherlands (cohort 5) using KASP (Kompetitive Allele Specific PCR, LGC, Manchester, UK) technology. [35] This technology was supplemented with in-house RT-PCR using Roche Assay-by-Design.

### 4.3. Data Analyses

#### 4.3.1. Susceptibility Analyses: Cohorts 1–3

Susceptibility to CT infection and the presence of the 4 SNPs was determined in cohorts 1–3. Cases were defined as tested PCR-positive for CT DNA during STI clinic visits. Controls were participants who had tested PCR-negative for CT during STI clinic visits. 

Genotype distributions were tested in all cohorts and between cohorts. In case genotype distribution did not differ significantly between cohorts (χ^2^ test), cohorts were combined to increase power. Analyses were performed for all 4 SNPs separately. Subsequently, multivariable logistic regression was performed on all 4 SNPs to evaluate whether SNPs would be predictive for the susceptibility of CT infections independent of other SNPs. The occurrence of SNPs in cases and controls was compared using χ^2^ tests, and risks of CT acquisition between different SNP distributions were described as odds ratios (OR) with 95% confidence interval (CI).

#### 4.3.2. Severity Analyses: Cohorts 1,2,4,5

The definition of severity of CT infection can be divided in two ways: (1) As an immediate symptomatic course of infection and (2) as late complications (i.e., pelvic inflammatory disease, ectopic pregnancy, and/or tubal factor infertility). Cohort 1 and 2 contained information regarding symptomatic course of infection, and cohort 4 and 5 contained information regarding late complication. The severity of CT infection and the presence of SNPs was assessed in 4 ways.

I: Investigating severity in terms of symptomatic CT infections versus asymptomatic CT infections, determining the presence of the SNPs in cohorts 1 and 2. Cases were defined as symptomatic CT positive women and controls as asymptomatic CT positive women.

II: Examining the presence of SNPs among CT positive women, comparing women positive for CT complications to women negative for CT complications. Cases were defined as women with a positive CT history and PID and/or ectopic pregnancy and/or TFI. In sensitivity analyses, PID was excluded in the definition to create a more specific outcome (due to heterogeneity in PID diagnosis). Cases were defined as women with a positive CT history and ectopic pregnancy and/or TFI. In cohort 4, TFI was defined as extensive peri-adnexal adhesions and/or distal occlusion of at least one tube, not attributable to abdominal pathology other than the genital tract infection (e.g., appendicitis) [33]. In Cohort 5, self-reported TFI was used. Controls were defined as women with a positive CT history without PID, ectopic pregnancy, and TFI and with at least one pregnancy of >20 weeks.

III: Performing trend analysis (i.e., a statistical procedure performed to evaluate hypothesized linear and nonlinear relationships between quantitative variables) to study the relationship between SNP occurrence and increased severity among cohorts 4 and 5. The hypothesis that the percentage of people carrying the risk genotypes would increase with increasing severity was tested. The groups, which were compared for trend, were arranged in order of severity: Fertile CT positive women (i.e., pregnant for at least once for >20 weeks), CT positive women with PID, CT positive women with ectopic pregnancy, and/or tubal factor infertility.

IV: It is well possible that SNPs in the different pathways do overlap and that women have multiple SNPs. Multiple SNPs can interact and, therefore, the result of having multiple SNPs might be different compared to just assessing all SNPs apart. To correct for this, we applied multivariable logistic regression to all four SNPs to evaluate whether the combination of SNPs would be predictive for the severity of CT infections. Analyses were performed on cohort 1 and 2 combined to assess the predictive value for symptomatology as well as on cohort 4 and 5 to assess the predictive value for long-term complications.

#### 4.3.3. Sensitivity Analysis

**Figure A1:**
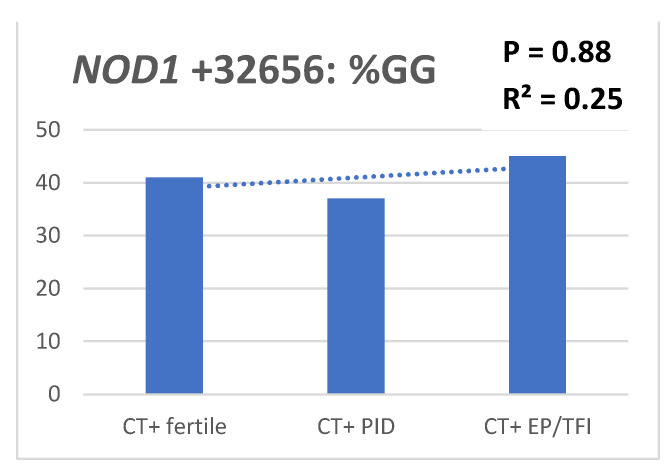
Sensitivity trend analysis using cohort (2) and a selection of cohort (1) comparing genotype distributions between women based on increased severity: (1) Fertile CT positive women. (2) CT positive women with PID. 3. CT positive women with ectopic pregnancy and/or tubal factor infertility. Percentage of genotypes as part of the total. Abbreviations: CT+, Chlamydia trachomatis positive; PID, pelvic inflammatory disease; EP, ectopic pregnancy; TFI, tubal factor infertility.

If the analysis involved women who previously had been tested for the described SNPs, a sensitivity analysis was performed excluding these women (Appendix A Table A1, Table A2, Table A3 and Table A4, Appendix A Figure A1). No sensitivity analysis regarding the susceptibility could be performed for *TLR2* and *CXCR5.* Cohort 1 had, in full, already been used to evaluate these SNPs. Sensitivity analysis using only cohort 2 proved impossible since this cohort existed only of CT positive women. Therefore, a different kind of sensitivity analysis was done comparing the CT positive women of cohort 2 with the CT negative women of cohort 1. In this way, a new comparison was made between cases that were never tested for the SNP and controls who were (Appendix A Table A1).

χ^2^ tests were used, and risks were described as odds ratios (OR) with 95% confidence interval (CI). *p* values < 0.05 were considered statistically significant. Analyses were performed using IBM SPSS Statistics. The regression coefficient (R^2^) for the trends was calculated using an ordinal scale in Microsoft Office Excel.

### 4.4. METC Approval

The act ‘Medical Research Involving Human Subjects’ (WMO, Dutch Law) states that anonymous spare human materials and data may be used for research purposes if the data are completely anonymized and not retrievable. Cohort 5 was approved by Medical Ethical Committee VU medical Center, Amsterdam the Netherlands (NL 51553.094.14/2015.903(A2019.336)). All participants provided informed consent for participation.

## Figures and Tables

**Table 1:** Genotype distribution by C. *trachomatis* (CT) status

Susceptibility		*IL-10*−1082			*NOD1* + 32656			*TLR2* + 2477			*CXCR5* + 10950		
		AA	AG	GG	TT	TGG	GGGG	GG	GA	AA	TT	TC	CC
Cohorts 1,2		92 (30.3%)	133 (43.8%)	79 (26.0%)	167 (54.9%)	121 (39.8%)	16 (5.3%)	280 (92.1%)	24 (7.9%)	0 (0%)	111 (36.5%)	146 (48.0%)	47 (15.5%)
CT	Negative	34 (34.7%)	38 (38.8%)	26 (26.5%)	52 (53.1%)	42 (42.9%)	4 (4.1%)	89 (90.8%)	9 (9.2%)	0 (0%)	35 (35.7%)	50 (51.0%)	13 (13.3%)
	Positive	58 (28.2%)	95 (46.1%)	53 (25.7%)	115 (55,8%)	79 (38.3%)	12 (5.8%)	191 (92.7%)	15 (7.3%))	0 (0%)	76 (36.9%)	96 (46.6%)	34 (16.5%)
Cohort 3		202 (28.6%)	365 (51.6%)	140 (19.8%)	427 (60.4%)	229 (32.4%)	5 (7.2%)	674 (95.3%)	30 (4.2%)	3 (0.4%)	246 (34.8%)	338 (47.8%)	123 (17.4%)
CT	Negative	184 (29.4%)	313 (50.0%)	129 (20.6%)	374 (59.7%)	204 (32.6%)	48 (7.7%)	596 (95.2%)	27 (4.3%)	3 (0.5%)	216 (34.5%)	300 (47.9%)	110 (17.6%)
	Positve	18 (22.2%)	52 (64.2%)	11 (13.6%)	53 (65.4%)	25 (30.9%)	3 (3.7%)	78 (96.3%)	3 (3.7%)	0 (0%)	30 (37.0%)	38 (46.9%)	13 (16.0%)

Abbreviation: CT, Chlamydia trachomatis. No significant differences were found between any of the distributions for chlamydia positive and negative women.

**Table 2:** Genotype distribution by severity of infection.

**Severity**	*IL-10*–1082			*NOD1* + 32656			*TLR2* + 2477			*CXCR5* + 10950		
**Analysis I: Cohorts 1,2**	**AA**	**AG**	**GG**	**TT**	**TGG**	**GGGG**	**GG**	**GA**	**AA**	**TT**	**TC**	**CC**
CT + total	56 (27.9%)	94 (46.8%)	51 (25.4%)	112 (55.7%)	77 (38,3%)	12 (6.0%)	187 (93.0%)	14 (7.0%))	0 (0%)	74 (36.8%)	94 (46.8%)	33 (16.4%)
CT + AS	34 (29.6%)	51 (44.3%)	30 (26.1%)	72 (62.6%)	36 (31.3%)	7 (6.1%)	110 (95.7%)	5 (4.3%)	0 (0%)	42 (36.5%)	55 (47.8%)	18 (15.7%)
CT + S	22 (25.6%)	43 (45.7%)	21 (24.4%)	40 (46.5%)	41 (47.7%)	5 (5.8%)	77 (89.5%)	9 (10.5%)	0 (0%)	32 (37.2%)	39 (45.4%)	15 (17.4%)
*p* value	*p* for GG vs. A* = 0.79			*p* for *GG vs. TT = 0.02 **			*p* for *A vs. GG = 0.10			*p* for CC vs. T* = 0.74		
**Analysis II: Cohorts 4,5**	**AA**	**AG**	**GG**	**TT**	**TGG**	**GGGG**	**GG**	**GA**	**AA**	**TT**	**TC**	**CC**
Total	45 (25.9%)	73 (42.0%)	56 (32.2%)	102 (58.6%)	64 (36.8%)	8 (4.6%)	165 (94.8%)	7 (4.0%)	2 (1.1%)	63 (36.2%)	80 (46.0%)	31 (17.8%)
Controls	31 (26.7%)	53 (45.7%)	32 (27.6%)	69 (59.5%)	40 (34.5%)	7 (6.0%)	109 (94.0%)	5 (4.3%)	2 (1.7%)	44 (37.9%)	55 (47.4%)	17 (14.7%)
Cases	14 (24.1%)	20 (34.5%)	24 (41.4%)	33 (56.9%)	24 (41.4%)	1 (1.7%)	56 (96,6%)	2 (3,4%)	0 (0.0%)	19 (32.8%)	25 (43.1%)	14 (24.1%)
*p* value	*p* for GG vs. A* = 0.07			*p* for *GG vs. TT = 0.74			*p* for *A vs. GG = 0.47			*p* for CC vs. T* = 0.12		

*p* values based on chi-square, significant results are marked with ** Abbreviations: CT+, Chlamydia trachomatis positive; AS, asymptomatic; S, symptomatic.

**Table 4:** Crude and adjusted results for susceptibility to infection

Susceptibility		*IL10*: GG vs. A*	*NOD1*: *GG vs. TT	*TLR2:* *A vs. GG	*CXCR5*: *C vs. TT
Cohorts 1,2	OR crude	1.0 (95%CI: 0.6–1.7)	0.9 (95%CI: 0.6–1.4)	0.8 (95%CI: 0.3–1.8)	1.0 (95%CI: 0.6–1.6)
	*p* value crude	0.88	0.65	0.57	0.84
	OR MLR	1.0 (95%CI: 0.6–1.7)	0.9 (95%CI: 0.6–1.5)	0.8 (95%CI: 0.3–1.9	1.0 (95%CI: 0.6–1.6)
	*p* value MLR	0.90	0.69	0.60	0.85
Cohort 3	OR crude	0.6 (95%CI: 0.3–1.2)	0.8 (95%CI: 0.5–1.3)	0.8 (95%CI: 0.3–2.9)	0.9 (95%CI: 0.6–1.4)
	*p* value crude	0.14	0.33	0.79	0.65
	OR MLR	0.6 (95%CI: 0.3–1.2)	0.8 (95%CI: 0.5–1.3)	0.9 (95%CI: 0.3–2.9)	0.9 (95%CI: 0.6–1.5)
	*p* value MLR	0.15	0.32	0.82	0.66

Abbreviations: MLR, multivariable logistic regression.

**Table 3:** Crude and adjusted results for severity of infection.

Severity		*IL10*: GG vs. A*	*NOD1*: *GG vs. TT	*TLR2:* *A vs. GG	*CXCR5*: CC vs. *T
Cohorts 1,2	OR crude	0.9 (95%CI: 0.5−1.7)	1.9 (95%CI: 1.1−3.4)	2.6 (95%CI: 0.8−8.0)	1.1 (95%CI: 0.5−2.4)
	*p* value crude	0.79	0.02 **	0.10	0.73
	OR MLR	0.9 (95%CI: 0.5−1.7)	1.9 (95%CI: 1.1−3.4)	2.4 (95%CI: 0.8−7.5)	1.1 (95%CI: 0.5−2.4)
	*p* value MLR	0.68	0.03 **	0.14	0.79
Cohorts 4,5	OR crude	1.9 (95%CI: 1.0−3.6)	1.1 (95%CI: 0.6−2.1))	0.6 (95%CI: 0.1−2.8)	1.9 (95%CI: 0.8−4.1
	*p* value crude	0.07	0.74	0.47	0.12
	OR MLR	1.9 (95%CI: 0.9−3.6)	1.3 (95%CI: 0.6−2.4)	0.5 (95%CI: 0.1−2.7)	1.9 (95%CI: 0.8−4.2)
	*p* value MLR	0.07	0.50	0.43	0.13

Significant results are marked with ** Abbreviations: MLR, multivariable logistic regression.

**Table 5:** Description of the cohorts.

Cohort	Cohort Description	n	CT Determination	Chlamydia Outcome	DNA Isolation	Severity Determination
Cohort 1: Patients from STI outpatient clinic Amsterdam	Women under the age of 33. Collected from July 2001 to December 2004 to investigate the role of a *CD14* SNP in susceptibility to a CT infection [30].	192	Cervical swabs were used for CT DNA detection by PCR (COBAS AMPLICOR; Hoffman–La Roche, Basel, Switzerland [6].	Chlamydia negative n = 98 Chlamydia positive Symptomatic n = 42 Asymptomatic n = 52	DNA was isolated from PBMC using isopropanol isolation [30].	Women completed a questionnaire regarding their symptoms at that moment.
Cohort 2: Patients from STI outpatient clinic the Hague	Collected from January to October 2008 to investigate the differences in IgG response in reaction to an infection by CT serogroup B, Serogroup I or serogroup C [31]	112	Cervical, vaginal, and/or urethral swabs and urine specimens were used for CT detection via probe hybridization assays (pace2 assay, Genprobe) [31].	Chlamydia positive Symptomatic n = 44 Asymptomatic n = 63 Unknown n = 5	DNA was isolated from serum using Roche High Pure PCR Template Preparation kit.	Information about symptoms was collected at the STI clinic or at the Department of Obstetrics and Gynaecology.
Cohort 3: Patients from STI outpatient clinic South-Limburg	Women between 18 and 33 years old, originally used to investigate an association between susceptibility to a CT infection and specific mutations in the vitamin D metabolism [32]	707	CT status was assessed using Roche Cobas 4800 NAAT	Chlamydia negative n = 626 Chlamydia positive n = 81	DNA was isolated from serum with a Hamilton Starlight isolation robot.	NA
Cohort 4: Gynaecology cohort from the University Medical Center Groningen	This women were part of a subfertility cohort aiming to investigate the influence of a *HLA-A* SNP to the severity of a CT infection. We used only the women with laparoscopically confirmed TFI [33].	12	Serum was used for a CAT test (pELISA, Medac Diagnostika, Germany). [33]		DNA was isolated from serum using Roche High Pure PCR Template Preparation kit.	All had undergone laparoscopy
Cohort 5: Subset of Netherlands Chlamydia Cohort Study (NECCST) [34]	Long-term prospective cohort aiming to determine CT complication risk and risk factors among women. Data collection (questionnaires, swabs and blood samples) from 2008–2016 [34].	178	Chlamydia positivity was determined by either a self-reported chlamydia infection, a positive PCR-test outcome in the CSI study and/or the presence of CT IgG antibodies in serum	All positive Self-reported infection n = 164 and/or Positive PCR test n = 26 and/or Presence of CT IgG n = 53	DNA was isolated from buccal swabs, vaginal swabs, or urine samples using Roche High Pure PCR Template Preparation kit [34].	Women completed a questionnaire regarding long term complications. PID n = 42 And/or TFI n = 9 And/or Ectopic pregnancy n = 6

From all cohorts, only women from West-European ethnicity were included; Abbreviations: CD14, cluster of differentiation 14; PCR, polymerase chain reaction; PBMC, peripheral blood mononuclear cell; IgG, Immunoglobulin G; NAAT, nucleic acid amplification test; HLA-A, human leukocyte antigen-A; CAT, Chlamydia antibody test; PID, pelvic inflammatory disease; TFI, tubal factor infertility.

## Data Availability

The data presented in this study are available on request from the corresponding author. The data are not publicly available due to consortium data limitations.

## Appendix A

**Table A1:** Sensitivity analysis, Genotype distribution by C. *trachomatis* (CT) status.

Susceptibility		*NOD1* + 32656				*TLR2* + 2477			*CXCR5* + 10950		
Sensivisity Analysis		TT	TGG	GGGG		GG	GA	AA	TT	TC	CC
Cohort 1 (selection), 2		103 (53.6%)	79 (41.1%)	10 (5.2%)	Cohort 1 (negatives), 2	191 (91.1%)	19 (9.0%)	0 (0.0%)	77 (36.7%)	103 (49.0%)	30 (14.3%)
CT	Negative	20 (51.3%)	18 (46.2%)	1 (2.6%)	Negative	89 (90.8%)	9 (9.2%)	0 (0.0%)	35 (35.5%)	50 (51.0%)	13 (13.3%)
	Positive	83 (54.2%)	61 (39.9%)	9 (5.6%)	Positive	102 (91.1%)	10 (8.9%)	0 (0.0%)	42 (37.5%)	53 (47.3%)	17 (15.2%)

No significant differences were found between any of the distributions for chlamydia positive and negative women. Abbreviations: CT, Chlamydia trachomatis.

**Table A2:** Sensitivity analysis, genotype distribution by severity of infection.

Severity	*NOD1* + 32656					*TLR2* + 2477		
**Analysis I: Cohorts 1 (selection), 2**	**TT**	**TGG**	**GGGG**	**Analysis I: Cohort 2**		**GG**	**GA**	**AA**
CT + total	80 (54.1%)	59 (39.9%)	9 (6.1%)	CT + total		98 (91.6%)	9 (8.4%))	0 (0%)
CT + AS	55 (61.1%)	29 (32.2%)	6 (6.7%)	CT + AS		60 (95.2%)	3 (4.8%)	0 (0%)
CT + S	25 (43.1%)	30 (51.7%)	3 (5.2%))	CT + S		38 (86.4%)	6 (13.6%)	0 (0%)
*p* value	*p* for *GG vs. TT = 0.03 **			*p* value		*p* for *A vs. GG = 0.08		
**Analysis II: Cohort 5**	TT	TGG	GGGG					
Total	97 (59.9%)	57 (35.2%)	8 (4.9%)					
Controls	69 (59.5%)	40 (34.5%)	7 (6.0%)					
Cases	28 (60.9%)	17 (37.0%)	1 (2.2%)					
*p* value	for *GG vs. TT = 0.87							

*p* values based on chi-square, significant results are marked with ** Abbreviations: CT+, Chlamydia trachomatis positive; AS, asymptomatic; S, symptomatic.

**Table A3:** Sensitivity analysis, crude and adjusted results for susceptibility to infection.

Susceptibility		*NOD1*: *GG vs. TT
Cohorts	OR crude	0.9 (95%CI: 0.4−1.8)
1 (selection), 2	*p* value crude	0.74
	OR MLR	0.9 (95%CI: 0.4−1.9)
	*p* value MLR	0.79

Abbreviations: MLR, multivariable logistic regression.

**Table A4:** Sensitivity analysis, crude and adjusted results for severity infection.

Severity		*NOD1*: *GG vs. TT			*TLR2:* *A vs. GG
Cohorts	OR crude	2.1 (95%CI: 1.1−4.1)	Cohort 2	OR crude	3.2 (95%CI: 0.7−13.4)
1 (selection), 2	*p* value crude	0.03 **		*p* value crude	0.10
	OR MLR	2.0 (95%CI: 1.0−4.0)		OR MLR	3.1 (95%CI: 0.7−13.1)
	*p* value MPL	0.04 **		*p* value MPL	0.13
Cohort 5	OR crude	1.1 (95%CI: 0.5−2.1)			
	*p* value crude	0.87			
	OR MLR	1.0 (95%CI: 0.5−2.0)			
	*p* value MLR	0.91			

Significant results are marked with **, Abbreviations: MLR, multivariable logistic regression.

## References

[1] Lal J.A., Malogajski J., Verweij S.P., De Boer P., Ambrosino E., Brand A., Ouburg S., Morre S.A. (2013). Chlamydia trachomatis infections and subfertility: Opportunities to translate host pathogen genomic data into public health. Public Health Genom..

[2] WHO Sexually Transmitted Infections (STIs). http://www.who.int/mediacentre/factsheets/fs110/en/.

[3] Staritsky L., van Aar F., Visser M., op de Coul E., Heijne J., Götz H., Nielen M., van Sighem A., van Benthem B. (2020). Sexually transmitted infections in the Netherlands in 2019. Seksueel Overdraagbare Aandoeningen in Nederland in 2019.

[4] Black C.M. (1997). Current methods of laboratory diagnosis of Chlamydia trachomatis infections. Clin. Microbiol. Rev..

[5] Den Hartog J.E., Ouburg S., Land J.A., Lyons J.M., Ito J.I., Pena A.S., Morre S.A. (2006). Do host genetic traits in the bacterial sensing system play a role in the development of Chlamydia trachomatis-associated tubal pathology in subfertile women?. BMC Infect. Dis..

[6] Spaargaren J. (2006). A Multidisciplinary Approach to the Study of Chlamydia Trachomatis Infections: Female Urogenital and Male Anorectal Infections.

[7] den Hartog J.E., Lyons J.M., Ouburg S., Fennema J.S., de Vries H.J., Bruggeman C.A., Ito J.I., Pena A.S., Land J.A., Morre S.A. (2009). TLR4 in Chlamydia trachomatis infections: Knockout mice, STD patients and women with tubal factor subfertility. Drugs Today.

[8] Hoenderboom B.M., van Benthem B.H.B., van Bergen J., Dukers-Muijrers N., Gotz H.M., Hoebe C., Hogewoning A.A., Land J.A., van der Sande M.A.B., Morre S.A. (2019). Relation between Chlamydia trachomatis infection and pelvic inflammatory disease, ectopic pregnancy and tubal factor infertility in a Dutch cohort of women previously tested for chlamydia in a chlamydia screening trial. Sex. Transm. Infect..

[9] Bailey R.L., Natividad-Sancho A., Fowler A., Peeling R.W., Mabey D.C., Whittle H.C., Jepson A.P. (2009). Host genetic contribution to the cellular immune response to Chlamydia trachomatis: Heritability estimate from a Gambian twin study. Drugs Today.

[10] Morre S.A., Karimi O., Ouburg S. (2009). Chlamydia trachomatis: Identification of susceptibility markers for ocular and sexually transmitted infection by immunogenetics. FEMS Immunol. Med. Microbiol..

[11] Mascellino M.T., Boccia P., Oliva A. (2011). Immunopathogenesis in Chlamydia trachomatis Infected Women. ISRN Obstet. Gynecol..

[12] Broeze K.A., Opmeer B.C., Coppus S.F., Van Geloven N., Den Hartog J.E., Land J.A., Van der Linden P.J., Ng E.H., Van der Steeg J.W., Steures P. (2012). Integration of patient characteristics and the results of Chlamydia antibody testing and hysterosalpingography in the diagnosis of tubal pathology: An individual patient data meta-analysis. Hum. Reprod..

[13] Darville T., O’Neill J.M., Andrews C.W., Nagarajan U.M., Stahl L., Ojcius D.M. (2003). Toll-like receptor-2, but not Toll-like receptor-4, is essential for development of oviduct pathology in chlamydial genital tract infection. J. Immunol..

[14] Verweij S.P., Karimi O., Pleijster J., Lyons J.M., de Vries H.J., Land J.A., Morre S.A., Ouburg S. (2016). TLR2, TLR4 and TLR9 genotypes and haplotypes in the susceptibility to and clinical course of Chlamydia trachomatis infections in Dutch women. Pathog. Dis..

[15] Brankovic I., van Ess E.F., Noz M.P., Wiericx W.A., Spaargaren J., Morre S.A., Ouburg S. (2015). NOD1 in contrast to NOD2 functional polymorphism influence Chlamydia trachomatis infection and the risk of tubal factor infertility. Pathog. Dis..

[16] Karimi O., Jiang J., Ouburg S., Champion C., Khurana A., Liu G., Poya H., Freed A., Pleijster J., Rosengurt N. CXCR5 Regulates Chlamydia Tubal Pahtology in Mice and Humans; 2011. https://research.vu.nl/files/42210838/chapter%2005.pdf.

[17] Ohman H., Tiitinen A., Halttunen M., Lehtinen M., Paavonen J., Surcel H.M. (2009). Cytokine polymorphisms and severity of tubal damage in women with Chlamydia-associated infertility. J. Infect. Dis..

[18] Ho D.S.W., Schierding W., Wake M., Saffery R., O’Sullivan J. (2019). Machine Learning SNP Based Prediction for Precision Medicine. Front. Genet..

[19] Zou Y., Lei W., He Z., Li Z. (2016). The role of NOD1 and NOD2 in host defense against chlamydial infection. FEMS Microbiol. Lett..

[20] Kinnunen A.H., Surcel H.M., Lehtinen M., Karhukorpi J., Tiitinen A., Halttunen M., Bloigu A., Morrison R.P., Karttunen R., Paavonen J. (2002). HLA DQ alleles and interleukin-10 polymorphism associated with Chlamydia trachomatis-related tubal factor infertility: A case-control study. Hum. Reprod..

[21] Sabat R., Grutz G., Warszawska K., Kirsch S., Witte E., Wolk K., Geginat J. (2010). Biology of interleukin-10. Cytokine Growth Factor Rev..

[22] Scapini P., Lamagna C., Hu Y., Lee K., Tang Q., DeFranco A.L., Lowell C.A. (2011). B cell-derived IL-10 suppresses inflammatory disease in Lyn-deficient mice. Proc. Natl. Acad. Sci. USA.

[23] Moore K.W., de Waal Malefyt R., Coffman R.L., O’Garra A. (2001). Interleukin-10 and the interleukin-10 receptor. Annu. Rev. Immunol..

[24] Turner D.M., Williams D.M., Sankaran D., Lazarus M., Sinnott P.J., Hutchinson I.V. (1997). An investigation of polymorphism in the interleukin-10 gene promoter. Eur. J. Immunogenet..

[25] Ohman H., Tiitinen A., Halttunen M., Birkelund S., Christiansen G., Koskela P., Lehtinen M., Paavonen J., Surcel H.M. (2006). IL-10 polymorphism and cell-mediated immune response to Chlamydia trachomatis. Genes Immun..

[26] Gibson A.W., Edberg J.C., Wu J., Westendorp R.G., Huizinga T.W., Kimberly R.P. (2001). Novel single nucleotide polymorphisms in the distal IL-10 promoter affect IL-10 production and enhance the risk of systemic lupus erythematosus. J. Immunol..

[27] Wang C., Tang J., Geisler W.M., Crowley-Nowick P.A., Wilson C.M., Kaslow R.A. (2005). Human leukocyte antigen and cytokine gene variants as predictors of recurrent Chlamydia trachomatis infection in high-risk adolescents. J. Infect. Dis..

[28] Lorenz E., Mira J.P., Cornish K.L., Arbour N.C., Schwartz D.A. (2000). A novel polymorphism in the toll-like receptor 2 gene and its potential association with staphylococcal infection. Infect. Immun..

[29] Genome of the Netherlands C. (2014). Whole-genome sequence variation, population structure and demographic history of the Dutch population. Nat. Genet..

[30] Ouburg S., Spaargaren J., den Hartog J.E., Land J.A., Fennema J.S., Pleijster J., Pena A.S., Morre S.A., Consortium I. (2005). The CD14 functional gene polymorphism -260 C>T is not involved in either the susceptibility to Chlamydia trachomatis infection or the development of tubal pathology. BMC Infect. Dis..

[31] Verweij S.P., Bax C.J., Quint K.D., Quint W.G., van Leeuwen A.P., Peters R.P., Oostvogel P.M., Mutsaers J.A., Dorr P.J., Pleijster J. (2009). Significantly higher serologic responses of Chlamydia trachomatis B group serovars versus C and I serogroups. Drugs Today.

[32] Lanjouw E., Brankovic I., Pleijster J., Spaargaren J., Hoebe C.J., van Kranen H.J., Ouburg S., Morre S.A. (2016). Specific polymorphisms in the vitamin D metabolism pathway are not associated with susceptibility to Chlamydia trachomatis infection in humans. Pathog. Dis..

[33] Jansen M.E., Brankovic I., Spaargaren J., Ouburg S., Morre S.A. (2016). Potential protective effect of a G > A SNP in the 3’UTR of HLA-A for Chlamydia trachomatis symptomatology and severity of infection. Pathog. Dis..

[34] Hoenderboom B.M., van Oeffelen A.A., van Benthem B.H., van Bergen J.E., Dukers-Muijrers N.H., Gotz H.M., Hoebe C.J., Hogewoning A.A., van der Klis F.R., van Baarle D. (2017). The Netherlands Chlamydia cohort study (NECCST) protocol to assess the risk of late complications following Chlamydia trachomatis infection in women. BMC Infect. Dis..

[35] He C., Holme J., Anthony J. (2014). SNP genotyping: The KASP assay. Methods Mol. Biol..

